# Development of a prognostic composite cytokine signature based on the correlation with nivolumab clearance: translational PK/PD analysis in patients with renal cell carcinoma

**DOI:** 10.1186/s40425-019-0819-2

**Published:** 2019-12-11

**Authors:** Rui Wang, Junying Zheng, Xiao Shao, Yuko Ishii, Amit Roy, Akintunde Bello, Richard Lee, Joshua Zhang, Megan Wind-Rotolo, Yan Feng

**Affiliations:** Bristol-Myers Squibb, Route 206 & Province Line Road, Princeton, NJ 08543 USA

**Keywords:** Clearance, Translational PK/PD analysis, Nivolumab, Cytokine, Composite signature, Renal cell carcinoma

## Abstract

**Background:**

Although several therapeutic options for patients with renal cell carcinoma (RCC) have been approved over recent years, including immune checkpoint inhibitors, considerable need remains for molecular biomarkers to assess disease prognosis. The higher pharmacokinetic (PK) clearance of checkpoint inhibitors, such as the anti–programmed death-1 (PD-1) therapies nivolumab and pembrolizumab, has been shown to be associated with poor overall survival (OS) across several tumor types. However, determination of PK clearance requires the collection and analysis of post-treatment serum samples, limiting its utility as a prognostic biomarker. This report outlines a translational PK-pharmacodynamic (PD) methodology used to derive a baseline composite cytokine signature correlated with nivolumab clearance using data from three clinical trials in which nivolumab or everolimus was administered.

**Methods:**

Peripheral serum cytokine (PD) and nivolumab clearance (PK) data from patients with RCC were analyzed using a PK-PD machine-learning model. Nivolumab studies CheckMate 009 (NCT01358721) and CheckMate 025 (NCT01668784) (*n* = 480) were used for PK-PD analysis model development and cytokine feature selection (training dataset). Validation of the model and assessment of the prognostic value of the cytokine signature was performed using data from CheckMate 010 (NCT01354431) and the everolimus comparator arm of CheckMate 025 (test dataset; *n* = 453).

**Results:**

The PK-PD analysis found a robust association between the eight top-ranking model-selected baseline inflammatory cytokines and nivolumab clearance (area under the receiver operating characteristic curve = 0.7). The predicted clearance (high vs low) based on the cytokine signature was significantly associated with long-term OS (*p* < 0.01) across all three studies (training and test datasets). Furthermore, cytokines selected from the model development trials also correlated with OS of the everolimus comparator arm (*p* < 0.01), suggesting the prognostic nature of the composite cytokine signature for RCC.

**Conclusions:**

Here, we report a PK-PD translational approach to identify a molecular prognostic biomarker signature based on the correlation with nivolumab clearance in patients with RCC. This composite biomarker signature may provide improved prognostic accuracy of long-term clinical outcome compared with individual cytokine features and could be used to ensure the balance of patient randomization in RCC clinical trials.

## Introduction

Renal cell carcinoma (RCC) accounts for approximately 3% of all adult cancers and about 90% of renal malignancies [1]. Patients with localized tumors typically undergo surgical resection, while systemic treatment is utilized for those with metastatic disease or who have relapsed after local therapy [1]. Therapeutic options for patients with advanced RCC have expanded rapidly over the past decade. Prior to the approval of vascular endothelial growth factor (VEGF) and mammalian target of rapamycin (mTOR) inhibitors, cytokine therapies such as high-dose interleukin 2 (IL-2) were the main treatment choice for advanced RCC [2, 3].

The responsiveness of kidney cancer to immunotherapies is well recognized [2]. Although cytokine therapy was associated with treatment-related toxicities and relatively low efficacy in 10–20% of patients, it provided proof-of-concept for novel immunotherapy agents in patients with RCC [4, 5]. In recent years, the approval of drugs targeting the immune checkpoint programmed death-1 (PD-1) has led to a considerable improvement in the survival of patients with advanced RCC [2, 6, 7]. Despite this progress, there is a need for the development of prognostic biomarkers to identify patients with RCC who are likely to benefit from immunotherapies [8]. Peripheral factors, such as circulating cytokines, have been shown to function as potential prognostic indicators for outcome [9, 10]. Furthermore, the simplicity of evaluating circulating cytokines and the advantages associated with minimally invasive sample collection add to the attractiveness of utilizing peripheral factors for prognosis [11]. Although some studies have explored the association between individual cytokines and clinical outcome, however, no composite cytokine signature that is prognostic in RCC has been found.

Pharmacokinetic (PK) clearance of monoclonal antibody checkpoint inhibitors, such as anti–PD-1 therapies (e.g. nivolumab and pembrolizumab) and anti–cytotoxic T lymphocyte antigen-4 (CTLA-4) (e.g. ipilimumab), has been identified as a surrogate marker of overall survival (OS) in several tumor types, including melanoma and non-small cell lung cancer [12–14]. Higher clearance may be associated with increased catabolic metabolism and cancer-induced cachexia (as indicated by lower albumin and higher lactate dehydrogenase). Thus, clearance has been hypothesized to be a surrogate marker of overall disease status. In addition, the effects of clearance and exposure on OS appear to be independent in multivariable survival analysis [12–14]. Specifically, the exposure of nivolumab was not a significant covariate of OS, after taking into account the effects of nivolumab clearance and other covariates (e.g. Eastern Cooperative Oncology Group performance status, baseline albumin) in the multivariable survival analysis. Indeed, among all covariates evaluated in the analysis, clearance showed the strongest association with OS [11, 12].

Despite the potential for clearance as a surrogate marker, its practical use as a prognostic indicator is limited by the requirement for post-treatment PK sampling. The development of a machine-learning model to derive a baseline cytokine signature correlated with nivolumab clearance has been previously reported, and was shown to have prognostic value in patients with advanced melanoma [15]. This study presents an extension of the application of this translational PK-pharmacodynamic (PD) approach to identify a prognostic composite cytokine profile in RCC.

## Methods

### Patients and study design

The primary analyses derive from two clinical studies: 1) a phase I randomized dose-ranging trial of nivolumab in patients with previously treated or treatment-naive advanced or metastatic RCC (CheckMate 009 [NCT01358721]), and 2) a phase III study of nivolumab vs everolimus in patients with previously treated advanced or metastatic RCC (CheckMate 025 [NCT01668784]). Data from patients treated with nivolumab monotherapy (*n* = 480) from these two studies were used as the training dataset for development of the machine-learning model. In addition, patients treated with nivolumab in a phase II randomized dose-ranging study of RCC in the second-line setting, CheckMate 010 (NCT01354431), as well as the patients randomized to the comparator arm and treated with everolimus in CheckMate 025, were included in the model application (test dataset; *n* = 453). All patients provided voluntary written informed consent. Brief details on study treatment, schedule, and patient number for each dataset included in the analyses are provided in Table 1. Additional details regarding the study designs for each trial have been published [7, 16, 17].

**Table 1:** Summary of clinical studies for model development and test application

Study	Treatment	Dose and schedule	Patient number^a^ (total treated)	Analysis
CheckMate 009 (NCT01358721), phase I dose escalation	Nivolumab	0.3, 2, and 10.0 mg/kg, Q3W	*N* = 89 (91)	Training dataset
CheckMate 025 (NCT01668784), phase III	Nivolumab	3.0 mg/kg, Q2W	*N* = 391 (406)	Training dataset
	Everolimus	10.0 mg as a daily dose	*N* = 297 (397)	Test dataset
CheckMate 010 (NCT01354431), phase II dose ranging	Nivolumab	0.3, 2, and 10.0 mg/kg, Q3W	*N* = 156 (167)	Test dataset

^a^Patients missing cytokine or pharmacokinetics data were excluded from the training and test datasets of the machine-learning model. *Q2W* every 2 weeks, *Q3W* every 3 weeks

### Patient serum cytokine assay

Cytokines in patient serum samples collected at baseline prior to study treatment were measured using Luminex-based technology (CustomMAP panel by combining several multiplex human inflammatory MAP panels; Myriad RBM, Austin, TX).

### Machine-learning model

PK and PD associations were characterized using elastic net, a machine-learning algorithm widely used in biomarker research [18]. Nivolumab clearance (PK) and inflammatory cytokine panel (PD) data from CheckMate 009 and 025 were used as training datasets for model development (Table 1). Nivolumab clearance was estimated from population PK analysis using a linear two-compartment model [19]. The median of baseline nivolumab clearance from the training dataset (11.3 mL/h) was used to categorize patients as belonging to a high- or low-clearance group.

Elastic net, a regularized regression model, was used in model development [20]. It is an embedded feature selection method that performs the variable selection as part of the statistical learning procedure [18]. The elastic net model was then built upon the cytokine data, and model performance was evaluated via cross-validation (10 folds/10 repeats). A panel of cytokines was selected during the statistical learning process and only the identified important features with coefficient estimates greater than 0 from the elastic net algorithm were used in the subsequent analysis. The model was then tested on an independent dataset of nivolumab monotherapy from CheckMate 010 (Table 1). The area under the receiver operating characteristic curve (AUC-ROC) was used as a measure of the overall performance of the predictive model. The predicted clearance value of each patient was classified into a high or low group, and the probability threshold to define high vs low was set to where total false positives and total false negatives were equal (here positive class refers to low clearance). Kaplan–Meier plots were generated based on the OS of patients in the predicted high- and low-clearance groups. Log-rank tests were performed to assess the statistical difference. All modeling and analyses were performed using R software (version 3.4.1). Survival analysis was conducted using Survival (version 2.41–3) and survminer package (version 0.4.0).

## Results

### Overview of the translational PK-PD approach to select cytokine features

**Fig. 1:**
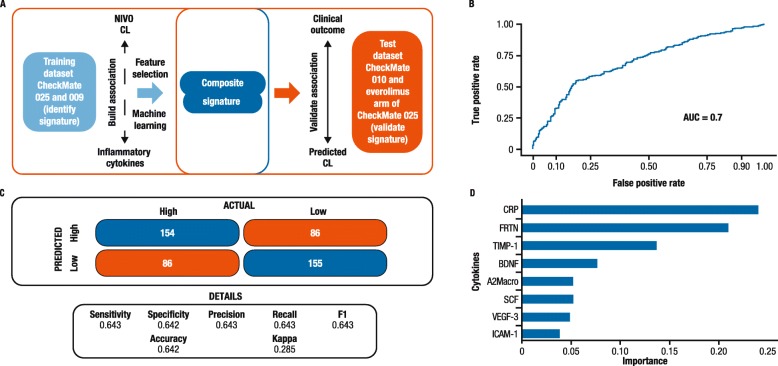
**a** Schematic overview of the machine-learning approach used to identify and then validate the composite prognostic biomarkers. **b** AUC-ROC analysis to show the performance of the machine-learning model (AUC = 0.7). **c** 2 × 2 analysis for actual clearance vs predicted clearance to show the accuracy of the model performance. **d** Selected cytokine features from the machine-learning model based on measured importance. Eight top-ranking cytokines were selected to form a composite signature: C-reactive protein (CRP), ferritin (FRTN), tissue inhibitor of metalloproteinase 1 (TIMP-1), brain-derived neurotrophic factor (BDNF), alpha 2-macroglobulin (A2Macro), stem cell factor (SCF), vascular endothelial growth factor-3 (VEGF-3), and intercellular adhesion molecule 1 (ICAM-1). *AUC-ROC* area under the receiver operating characteristic curve, *CL* clearance, *F1* harmonic mean of precision and recall, *NIVO* nivolumab

We have previously reported the development of a machine-learning model to establish a correlation between baseline cytokines and nivolumab clearance in melanoma [15]. Given that nivolumab clearance, a PK parameter, has been shown to be a surrogate prognostic marker of survival across multiple tumor types (e.g. melanoma and non-small cell lung cancer) [12–14], the aim was to determine if the same approach could be applied to RCC. The biomarker signatures were identified in a training dataset via translational PK-PD analysis and then validated in an independent dataset. The entire framework contains training dataset processing, model building, biomarker signature selection, and external validation in test dataset (Fig. 1a). First, the elastic net algorithm was introduced to build the association between baseline cytokines and clearance in patients from CheckMate 009 and 025 (training datasets; Table 1). The selected cytokine features were then validated in another independent test dataset (CheckMate 010; Table 1) to predict the clearance level (high vs low) of patients (Fig. 1a). Performance of the predictive model was evaluated by AUC-ROC analysis with an average AUC of 0.7 (Fig. 1b). The 2 × 2 confusion matrix analysis also demonstrated a relatively high accuracy of 0.64 (Fig. 1c), which confirmed good model performance and high concordance between actual clearance and the predicted clearance value generated from the model. As a result, the top eight inflammatory cytokine features were selected to form the composite signature according to the measured importance. The selected cytokines were C-reactive protein (CRP), ferritin (FRTN), tissue inhibitor of metalloproteinase 1 (TIMP-1), brain-derived neurotrophic factor (BDNF), alpha 2-macroglobulin (A2Macro), stem cell factor (SCF), vascular endothelial growth factor-3 (VEGF-3), and intercellular adhesion molecule 1 (ICAM-1) (Fig. 1d).

### Identification and validation of the composite cytokine signature

**Fig. 2:**
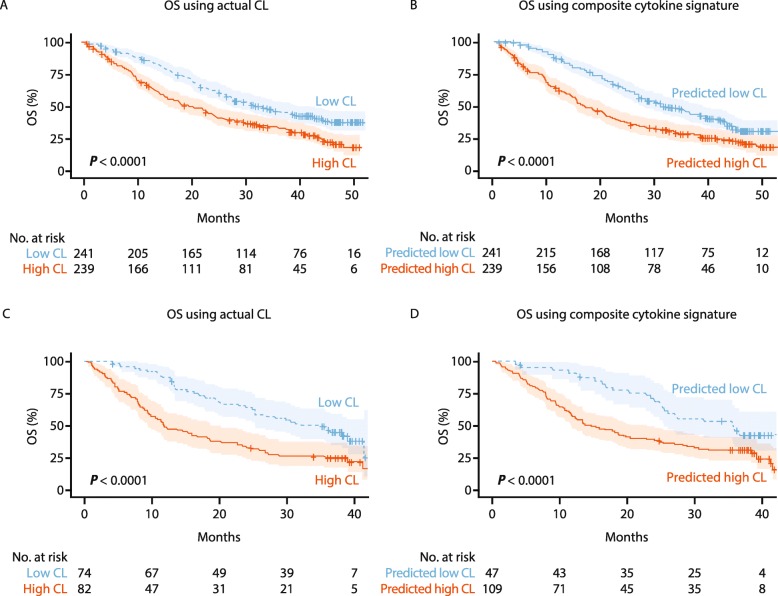
Evaluation of the composite cytokine signature in the training dataset (CheckMate 009 and 025) and validation of the signature in the test dataset (CheckMate 010) by comparing the outcome association from **a** actual nivolumab clearance in the training dataset; **b** predicted clearance using the composite cytokine signature in the training dataset; **c** actual nivolumab clearance in the test dataset; and **d** predicted clearance using the composite cytokine signature in the test dataset. *High CL* patients with high actual clearance, *low CL* patients with low actual clearance, *OS* overall survival, *predicted high CL* patients predicted to have high clearance from the cytokine signature, *predicted low CL* patients predicted to have low clearance from the cytokine signature

Calculated actual clearance was a robust predictor of OS in previously treated or treatment-naive patients with RCC (*p* < 0.0001, Fig. 2a). Based on clearance predicted from the composite cytokine signature, patients from CheckMate 009 and CheckMate 025 (training dataset) were classified into high and low clearance groups, and differences in OS between predicted low and high clearance groups were evaluated (Fig. 2b). The results demonstrated similar association between both actual and predicted high clearance with poor OS (higher risk of event and shorter OS). Patients with predicted low clearance had a significantly longer OS than those with high clearance (*p* < 0.0001, Fig. 2b).

To further validate the identified composite signature as a potential biomarker associated with efficacy, we applied it to an independent dataset from CheckMate 010. For this validation dataset, model performance demonstrated by the AUC-ROC curve was 0.71 and accuracy was 0.68. As shown in the Kaplan–Meier analyses, both actual and predicted clearance groups (high vs low) were significantly associated with OS (*p* < 0.01), whereby patients in the lower-clearance group had longer OS than patients with higher clearance (Fig. 2c and d). Our results suggest that the selected composite baseline cytokine profile was able to stratify patients into low- and high-risk groups and was significantly associated with OS in the independent test dataset (*p* < 0.01; Fig. 2d). The robust association of the identified composite signature with OS was observed in independent training and validation clinical studies.

### Exploring the prognostic value of the composite cytokine signature

**Fig. 3:**
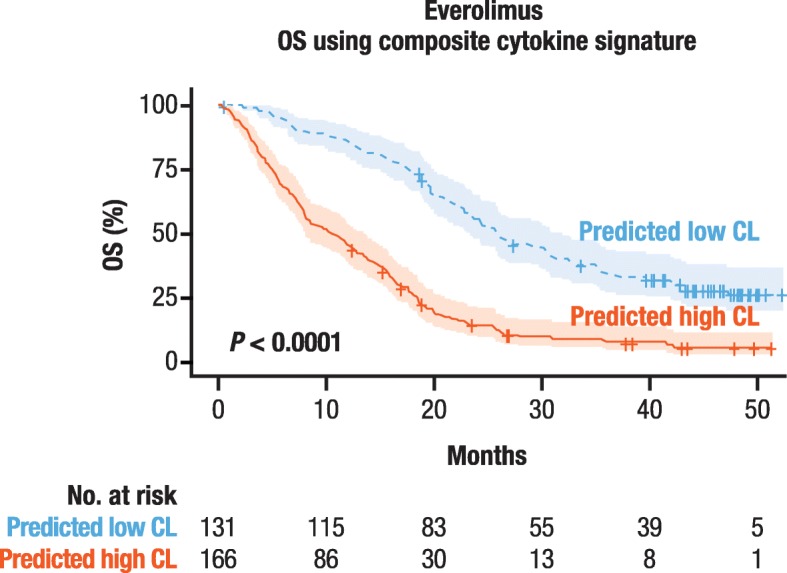
The predicted clearance of patients treated with everolimus (comparator arm of CheckMate 025), via the prognostic cytokine signature, was associated with OS. *OS* overall survival, *predicted high CL* patients predicted to have high clearance from the cytokine signature, *predicted low CL* patients predicted to have low clearance from the cytokine signature

To evaluate the prognostic value of the identified cytokine composite signature in patients with RCC, we used it to predict nivolumab clearance groups in patients randomized to everolimus in CheckMate 025. After categorization based on high or low clearance, Kaplan–Meier analysis was conducted to evaluate the association between predicted clearance group and OS. As shown in Fig. 3, a significant difference was observed between groups predicted to have high or low clearance (*p* < 0.0001), with patients with low clearance shown to have longer OS. It is important to point out that actual clearance data were not available for patients in the comparison cohort treated with everolimus, which highlights the additional value of applying the current approach to generate predicted clearance values. Taken together, these results confirm the prognostic role of the composite cytokine signature in patients with RCC, which is consistent with our previous observation that clearance has been shown to be strongly associated with OS in multivariable survival analyses [12–14].

## Discussion

In the present study and to our knowledge, this is the first time a composite cytokine signature containing eight cytokines selected by the machine-learning analysis based on the correlation with nivolumab clearance in RCC has been identified and validated. The identified signature was associated with RCC prognosis, regardless of treatment with nivolumab or everolimus, suggesting its potential utility as a novel independent prognostic tool. The strong association between OS and predicted clearance via the composite cytokine signature in everolimus-treated patients supported our hypothesis that clearance, potentially associated with patients’ overall disease status, could serve as a useful marker of long-term survival benefit.

Our data demonstrate that patients can be stratified based on the prognostic cytokine signature and the predicted high clearance value is significantly correlated with high risk, poor prognosis, and shorter OS. Therefore, the cytokine signature has the potential to be developed as a stratification factor in clinical trials in order to minimize imbalanced enrollment between experimental and comparator arms. Indeed, our data in advanced melanoma have demonstrated that the machine-learning approach could identify a prognostic composite cytokine signature that is strongly associated with OS in a specific disease [15]. Additionally, the observations across several RCC cohorts support the hypothesis that this PK-PD translational approach can be expanded to multiple indications for a broader application.

An increasing number of studies have shown that clearance of monoclonal anti–PD-1 antibody therapies, including nivolumab, might reflect patients’ overall disease status and thus could be utilized as a surrogate prognostic biomarker [14, 19, 21]. However, clearance values can only be derived from post-treatment PK assessment, which limits their clinical application. Therefore, the development of this baseline eight-cytokine prognostic signature for RCC, via correlation with clearance, is a highly practical way to utilize this robust association. Many of the cytokines identified have been previously reported to be individually associated with survival or treatment outcome in patients with RCC and other cancers. Normal baseline levels of CRP have been shown to predict longer progression-free survival and OS in patients with advanced RCC treated with sunitinib [22]. TIMP-1 may serve as a prognostic indicator for progression and metastasis in colon cancer [23]. In addition, studies have found that elevated VEGF levels were correlated with poor prognosis and disease progression in RCC [24]. Moreover, because the predictive power of individual cytokines is limited [25], the current multivariable approach resulting in tumor-specific composite signatures may provide improved prognostic accuracy for long-term clinical outcome. Notably, the previously reported composite cytokine signature in melanoma and the composite cytokine signature identified in the current study share several of the same individual cytokines, such as CRP, TIMP-1, and FRTN, but also include different cytokines. These differences may be due to tumor-specific disease characteristics and other patient-level characteristics. Comparative signature analyses and expansion of the approach in other tumor types is warranted.

In conclusion, our results suggest that the eight-cytokine signature identified is associated with survival and could serve as a clinically useful prognostic biomarker for patients with RCC. However, the mechanisms underlying the linkage between drug clearance and disease status of patients remains unconfirmed. Therefore, further investigation is warranted to verify the findings of these analyses, elucidate the potential molecular mechanisms, and develop a deeper understanding of the role of clearance in disease prognosis. Future work could include applying this novel PK-PD translational approach to identifying other types of biomarkers through genomic and proteomic analyses.

## Data Availability

The datasets generated and/or analyzed during the current study are not publicly available due to proprietary restrictions but are available from the corresponding author on reasonable request.
